# Recent advances in managing fecal incontinence

**DOI:** 10.12688/f1000research.15270.2

**Published:** 2019-08-23

**Authors:** Giovanna Da Silva, Anne Sirany

**Affiliations:** 1 Department of Colon and Rectal Surgery, Cleveland Clinic Florida, Weston, FL, 33331, USA

**Keywords:** Fecal incontinence, Cleveland Clinic Incontinence, Magnetic anal sphincter augmentation, Percutaneous nerve evaluation, Percutaneous tibial nerve stimulation, Sacral nerve neuromodulation.

## Abstract

Fecal incontinence (FI) is the uncontrolled passage of feces or gas in an individual who previously had control. The prevalence of the problem varies but can be as high as 50% of institutionalized individuals. The severity varies among individuals, but the negative impact on self-esteem and quality of life can have devastating effects. The goals of treatment are to decrease the frequency and severity of episodes as well as to improve quality of life. At present, several therapies, ranging from medical management to more invasive surgical interventions, are offered for the management of FI. In this article, we review the most recent advances in the management of FI.

## Introduction

Fecal incontinence (FI) is defined as the uncontrolled passage of feces or gas over at least one month’s duration in an individual who had previously achieved control ^1^. The severity of FI varies widely but can have significant negative impacts, including reduced quality of life, negative psychological effects, and associated social stigma ^2^. The etiology of FI may be multifactorial, and risk factors include old age, female sex, sphincter injury, obstetrical trauma, post-surgical complications, diarrhea, and constipation ^3, 4^. Prevalence rates vary but have been reported to range from 1.4 to 18% and up to 50% in institutionalized patients ^5^.

The goals of treatment are to decrease the frequency and severity of episodes and improve quality of life. The decision of which treatment to employ is based on the severity of symptoms and integrity of the anal sphincter. A continence scoring system such as the Cleveland Clinic Florida-Fecal Incontinence Score (CCF-FIS) is helpful in the evaluation of these patients ( Table 1). The American Society of Colon and Rectal Surgeons (ASCRS) Clinical Practice Guidelines recommend that first-line therapy for FI include dietary modifications, medical management with anti-diarrheal or fiber supplement (or both) to bulk the stool, and biofeedback exercises, which can lead to improvement in a significant portion of patients with mild FI. Adjuncts to these conservative measures have recently been reported and include the vaginal bowel control system (VBS) and anal plugs. Patients with more severe disease or sphincter defects will require more invasive procedures which can be categorized into methods that repair (sphincteroplasty), augment (injectables, radiofrequency remodeling, stem cell therapy, and posterior anal sling), or replace (adynamic muscle transfers, dynamic graciloplasty, artificial bowel sphincter, and magnetic anal sphincter) the anal sphincter or neuromodulate bowel function (sacral nerve neuromodulation [SNM] and posterior tibial nerve stimulation) or divert fecal transit (stoma and antegrade stoma procedure) ^6^. This article will focus on the most recent advances in the management of FI. Table 2 shows a list of the advancements in the area and whether or not there are available published randomized trials to support them.

**Table 1.** Cleveland Clinic Florida-Fecal Incontinence Score.

Type of incontinence	Frequency				
	Never	Rarely	Sometimes	Usually	Always
Solid	0	1	2	3	4
Liquid	0	1	2	3	4
Gas	0	1	2	3	4
Wears pad	0	1	2	3	4
Lifestyle alteration	0	1	2	3	4

Never, 0; Rarely, <1/month; Sometimes, <1/week, ≥1/month; Usually, <1/day, ≥1/week; Always, ≥1/day. Minimum score 0 (perfect continence), maximum score 20 (complete incontinence).

**Table 2.** Advancements in the treatment of fecal incontinence and the availability of published randomized trials.

Recent advance	Randomized trial results available	Comments
Anal insert	Yes	Total of 136 patients. Two studies compared plug versus no plug, and two studies compared different types of plugs. Data limited and incomplete ^7, 8^.
Vaginal bowel control system	No	
Stem cell therapy	Yes	The largest trial included 24 patients (12 in the treatment arm) and showed superior results with stem cell therapy ^17– 22^.
Anal sling	No	
Sacral nerve neuromodulation (SNM)	Yes	See below.
Percutaneous tibial nerve stimulation	Yes	Not better than placebo or SNM
Magnetic anal sphincter augmentation	Pending	

### Anal insertion device

**Figure 1.**
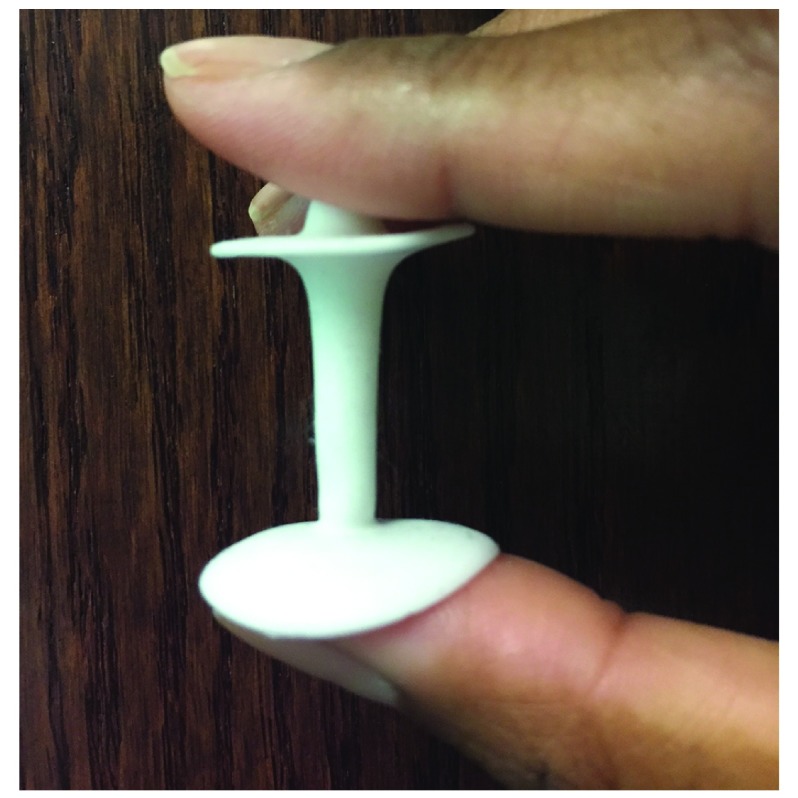
Renew insert device.

The use of anal plugs was described in the early 2000s. The concept is simple: the goal is to block the involuntary passage of stool from the anus. The device is self-inserted by patients and self-expelled or manually removed with a bowel movement. The data regarding the use of anal insertion devices for the treatment of FI are limited. A Cochrane Review in 2015 ^7^ found only four studies that met eligibility criteria and noted that plugs can be difficult to tolerate. In patients who are able to tolerate the device, there is limited evidence that plugs may be helpful in alleviating problems due to FI. The most recent device on the market, not included in the Cochrane Review, is a soft silicone anal insert ^8^. In a multicenter prospective study, 77% of the 73 patients who completed the study and 62% of the 91 intention-to-treat patients achieved at least a 50% reduction in incontinence frequency over a 12-week period. The device, however, was used for patients with moderate to severe FI. It is the authors’ opinion that patients with mild FI or leakage are most likely to benefit from this type of device ( Figure 1).

### Vaginal bowel control system

**Figure 2.**
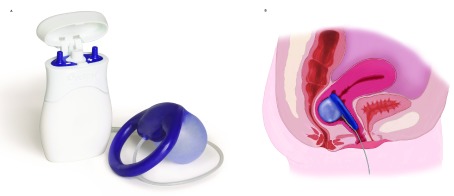
Vaginal bowel control system. The vaginal bowel control insert, folded and deflated for insertion (right) and inflated as it would sit in the vagina; the insert is inflated with a handheld pressure-regulated pump. Reprinted with permission from Pelvalon Inc. (Sunnyvale, CA, USA).

The VBS consists of a vaginal insert and a pressure-regulated pump introduced into the vagina ( Figure 2) *.* Patients are able to deflate the vaginal insert for defecation versus having to remove the device altogether, as is the case with the anal plug. In 2015, Richter *et al*. ^9^ reported the results of 61 women successfully fitted and treated with the VBS. At 1 month, 79% of patients demonstrated treatment success and 41% reported complete continence. At 3 months, 86% reported success and 45% reported complete continence. The VBS device was found to be easy to use and to have a high degree of efficacy and relatively few adverse effects. It is limited by the fact that not all women can be fitted with the device. In the study by Richter *et al*., only 54.5% of participants could be fitted with the device; however, once fitted, 96% found the device comfortable. More recently, the results of a multicenter, open-label trial including 73 patients with a mean CCF-FIS of 14.1 ± 12.15 showed a success rate of 72.6% at 3 months in the intention-to-treat analysis and of 84.1% in the per-protocol (patients able to keep the device) analysis. At 6 and 12 months, success remained high, improving to 90% and 94%, respectively, in the per-protocol population. There was significant improvement in the Fecal Incontinence Quality of Life score and the satisfaction rate reached over 94% at 12 months. This study showed that the VBS seemed to have a durable efficacy in patients who were successfully fitted and who had initial treatment response ^16^.

### Bulking agents

Injection of a bulking agent to augment the function of the anal sphincter for the treatment of FI was first described in 1993 ^11^. The ideal agent should be biocompatible, non-immunogenic, and small enough to inject and have minimal migration of the agent ^1^. Multiple agents have been proposed over the years. A Cochrane Review in 2010 ^12^ concluded that there was little evidence to support the use of bulking agents in the treatment of FI. In 2011, the US Food and Drug Administration (FDA) approved a non-animal-stabilized hyaluronic acid dextranomer gel for submucosal injection (NASHA Dx) for the treatment of FI. The largest series to date compared injection of NASHA Dx versus sham injection in a randomized, sham-controlled trial ^13^. Again, the primary endpoint was a more than 50% reduction in the number of incontinence episodes and an increase in incontinence-free days. Eighty percent of patients in the NASHA Dx group actually had a second injection within 1 month after no improvement with the first injection. Those in the NASHA Dx group had a 52% reduction compared with 31% in the sham group ( *P* = 0.0089). Despite this reduction, there was no significant difference in the FI scores between the groups at 2 months. In the published long-term follow-up ^14^, the reduction in incontinence episodes was sustained at 12 (57%) and 36 (52%) months and the mean CCF-FIS was lower at 36 months. Despite improvement in FI, patients still had what is considered important FI (CCF-FIS of 11). NASHA Dx may be more beneficial for patients with minor, passive FI such as those with limited internal sphincter injury rather than for patients with more severe FI as in the case of most of the studies in the literature. Its final role in the treatment of FI, however, remains uncertain as a randomized trial comparing NASHA Dx with biofeedback did not show difference between the treatments ^15^. A new bulking agent consisting of copolymer particle of polyacrylate-polyalcohol immersed in a carrier of glycerol and saline was described in 2015 ^16^. In total, 58 patients were enrolled in the study, which followed patients up to 36 months post-injection. Sixty percent of patients were successfully treated (with 50% more improvement in the CCF-FIS), although only 41.5% of patients achieved long-term follow-up to 36 months. Further research is needed to confirm these results and demonstrate the efficacy and safety of this new substance in the treatment of FI. Injectable agents are contraindicated in patients with inflammatory bowel disease, rectocele, prolapse, previous pelvic irradiation, or anorectal malformations.

### Stem cell therapy

Stem cell therapy has been evaluated for use in a variety of clinical settings such as cardiovascular, hematological, neurological, digestive, and trauma-associated conditions. The most commonly used stem cells are hematopoietic stem cells, mesenchymal stem cells, and adipose-derived stem cells ^17^. The use of stem cells for the treatment of FI has been postulated as a means of providing recovery of sphincter function through regeneration of damaged striated sphincter muscle and allowing reinnervation of newly formed myofibers ^18^. The early literature on stem cells in FI was based on animal models demonstrating the safety and efficacy of stem cells for FI ^18– 20^. The first report of stem cell therapy and FI was by Lorenzi *et al*. in 2008 ^20^. In this animal model of FI, the authors demonstrated that bone marrow–derived mesenchymal stem cell injection improved muscle regeneration and increased contractile function of anal sphincters after injury and repair.

The first study in humans was an observational study by Frudinger *et al*. ^21^. They injected autologous myoblasts into the external anal sphincter in 10 women with lesions that had not been operated on and were refractory to conservative management. The authors demonstrated improved Wexner Incontinence Scores and quality of life at 12 months but did not demonstrate any significant physiological change to account for these improvements. These results were sustained at 5-year follow-up ^22^. There were no significant side effects or adverse events noted in any patient. In 2015, Bisson *et al*. ^18^ published a proof-of-concept study evaluating the injection of myoblasts into the anal sphincter of rats. The authors demonstrated sustained increase in sphincter pressures after injection. The follow-up, phase II, randomized, double-blind, placebo-controlled study in humans was published in 2018 ^23^. In total, 24 patients were enrolled: 12 in the treatment arm and 12 in the control arm. At 6 months, both groups demonstrated reduction in the Cleveland Clinic Incontinence (CCI) score, but at 12 months, the treatment group continued to show improvement in the CCI score whereas the control arm did not. No severe adverse events were reported. Injection of stem cells appears to be safe, does not have major adverse side effects, and provides clinical benefit in the treatment of FI. To date, there have been no phase III trials comparing stem cell therapy with the SNM and this option of treatment is not currently approved by the FDA in the US.

### Anal sling

Parks *et al*. emphasized the importance of the anorectal angle in the maintenance of continence ^24^. Based on this theory, the use of an artificial sling to support the posterior rectal wall has been developed for the treatment of FI ^25– 27^. Most recently, Mellgren *et al*. ^27^ reported on the 1-year data of a prospective, multicenter study involving 152 patients at 14 centers in the US using the Transobturator Posterior Anal Sling (TOPAS) (American Medical Systems, Minnetonka, MN, USA). The mesh is placed behind the anorectum via two small incisions in the buttocks, and each arm of the mesh exits through the obturator foramen ^28^. The mechanism of action is not exactly clear but is thought to support the puborectalis muscle and reinforce the anorectal angle. At 12 months, 69% of patients met the requirement for treatment success (at least 50% reduction in the FI episodes from baseline to 12 months post-operatively) and 19% reported complete continence. The posterior anal sling has not been compared with other treatment options for FI, such as SNM, and is not available in the US.

### Sacral nerve neuromodulation

**Figure 3.**
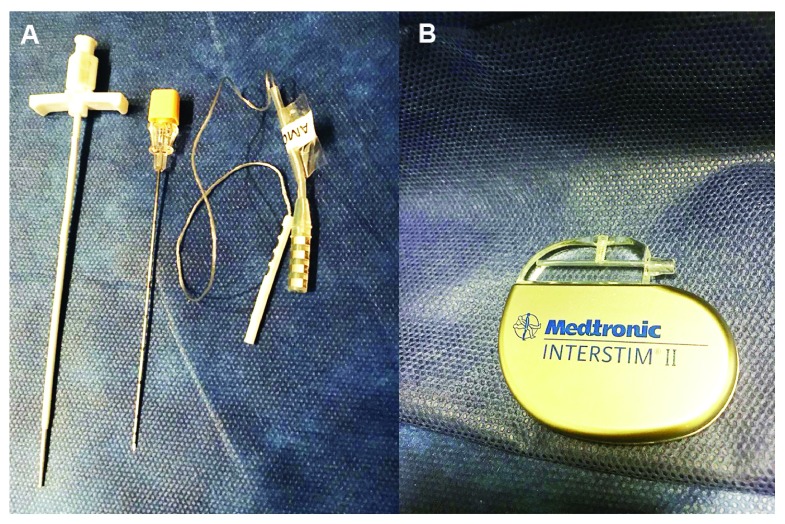
Sacral nerve neuromodulation. Courtesy of Lucia Oliveira, Rio de Janeiro, Brazil.

SNM was approved for use in the management of FI by the FDA in 2011 ( Figure 3). SNM works by electrical stimulation of the sacral nerve roots, producing anal sphincter augmentation and modulation of spinal/supraspinal pathways ^29^. Wexner *et al*. ^30^ demonstrated the efficacy of this device in the treatment of FI in a prospective, multicenter study in the US. Success was defined as at least 50% reduction of incontinent episodes per week over 12 weeks in at least 50% of patients. The authors demonstrated an 83% therapeutic success at 12 months, and 41% of patients achieved 100% continence. In long-term follow-up at 3 years ^31^, 83 patients were evaluated, success was demonstrated in 86% of patients, and 40% achieved complete continence. The most common reported adverse events were implant site pain (28%), parasthesias (15%), change in sensation of stimulation (12%), and infection (10%). The majority of adverse events (67%) occurred within the first year of implant. Of patients with infections, only six required surgical intervention: five device explants and one device replacement. A pooled analysis of all studies to date found that 69 to 83% of patients achieved success ^32^.

For many years, the only option for patients with a sphincter defect of up to 120 degrees was sphincter repair, but results are known to deteriorate over time. Soon after its advent, SNM was also demonstrated to be effective in patients with FI in the setting of sphincter injury. Brouwer and Duthie ^33^ found that SNM provided significant improvement of FI even in the presence of a sphincter defect or pudendal neuropathy. In the largest study that evaluated this, 91 patients with no sphincter defect were compared with 54 patients with imaging-documented external defect. There was no significant difference in the comparison of baseline and 12-month CCF-FIS between the two groups. The ASCRS strongly recommends SNM as a first-line surgical option of FI in patients with or without sphincter defects.

SNM is a two-stage procedure involving a test phase followed by permanent implantation. The test phase is predictive of success with the permanent device. The test phase can be completed with placement of an external stimulator in the operating room (OR) and a subsequent trip to the OR for permanent implantation or by percutaneous nerve evaluation (PNE) in the office setting. PNE is commonly used among surgeons in Europe, whereas a two-stage procedure is used in the US. Rice *et al*. ^34^ compared PNE with the traditional staged approached used in the US. They demonstrated that success was similar between the two approaches (90.2% for staged and 82.2% for PNE, *P* = 0.36), and the 3-month CCF-FIS was not significantly different (4.4 versus 4.1, *P* = 0.74). However, they did find that the infection rate was higher in the staged approach (10.5% versus 0%, *P* ≤0.005). On the other hand, a prospective randomized trial comparing one-stage to two-stage SNM for urinary incontinence found no difference in infection rates and significantly higher failure rates in patients undergoing the one-stage procedure ^35^. A systematic review by Maeda *et al*. ^36^ reported that the most common complication after PNE was lead displacement in 5.3% of patients and an overall complication rate of 6.4%, although 60% of studies did not report any complications related to PNE.

### Percutaneous tibial nerve stimulation

Although SNM is the first-line surgical intervention for FI, it does currently require two trips to the OR and has associated high equipment costs. Alternatives to SNM involving neuromodulation have recently been investigated. Percutaneous tibial nerve stimulation (PTNS) is thought to cause similar changes in anorectal neuromuscular function as SNM because of the shared sacral segmental innervation ^37^. This treatment is not currently available in the US. PTNS is a minimally invasive technique that can be performed in the outpatient setting. The initial data come from several case series, but more recently, two randomized controlled trials have evaluated this therapy ^37, 38^, one with placebo and one with SNM ^39^. In the CONFIDeNT trial ^37^, 227 patients were randomly assigned to PTNS versus sham PTNS over a 12-week period. There was no significant clinical benefit of PTNS over sham stimulation in the treatment of FI as measured by a more than 50% reduction in the number of FI episodes, although the absolute number of FI episodes per week was significantly reduced in the PTNS group.

An additional randomized control trial by Van der Wilt *et al*. ^38^ compared PTNS with sham stimulation. In total, 59 patients were included in the trial. The authors demonstrated a statistically significant reduction in the median and mean number of FI episodes of severity over 9 weeks. However, this was not clinically significant when response to treatment was based on a more than 50% reduction in FI episodes per week. A third trial ^39^ compared 17 patients undergoing SNM with 16 patients receiving PTNS and showed slightly superior results with SNM in the short term. All trials demonstrate that PTNS may be beneficial for some patients who have failed conservative management, but the trials have not established PTNS as a clinically meaningful treatment option, especially compared with SNM.

### Magnetic anal sphincter augmentation

A few methods and devices have been developed for patients who have severe FI or major sphincter disruption and who have failed previous treatments. These have included dynamic graciloplasty and artificial bowel sphincter. Patients who have retained the devices experienced significant improvement, but owing to high rates of complications and explantations, these procedures are no longer available.

The magnetic anal sphincter augmentation (MAS) is a newer therapeutic option for patients with FI and was approved by the FDA in 2015 as a humanitarian use device. The MAS device is a ring of magnetic beads which is surgically implanted around the anal sphincter to reinforce weakened sphincter muscles. Several studies have reported promising short-term outcomes ^40, 41^. A 2017 publication by Sugrue *et al*. ^42^ reported on the long-term overall outcomes at a median follow-up of 5 years. A total of 35 patients underwent implantation of the device. Therapeutic success rates were reported as 63% at 1 year, 66% at 3 years, and 53% at 5 years. The device was explanted in seven patients because of major adverse events. In total, 30 adverse events ranging from pain to device erosion were reported in 20 patients. The authors state that the majority (73%) of these were minor events and require little to no intervention and occurred during the first year after implantation. Two ongoing randomized controlled trials are comparing MAS with SNM and hopefully will shed light as to where MAS fits into the treatment algorithm of FI ^43, 44^.

## Conclusions

At present, several therapies, ranging from medical management to more invasive surgical intervention, are offered for the management of FI. Not all therapies are appropriate for every patient. Therefore, it is important to select the correct therapy for the specific patient. For patients with mild FI, we recommend conservative and non-invasive adjuncts. Patients with minor leakage secondary to an internal sphincter defect may benefit from injectables. Those with moderate to severe FI should be evaluated for SNM. Lastly, those with severe FI or those who have failed several prior therapies may be good candidates for a trial of MAS prior to committing to a permanent stoma.

## Abbreviations

ASCRS, American Society of Colon and Rectal Surgeons; CCF-FIS, Cleveland Clinic Florida-Fecal Incontinence Score;; FDA, CCI, Cleveland Clinic Incontinence; US Food and Drug Administration; FI, fecal incontinence; MAS, magnetic anal sphincter augmentation; NASHA Dx, non-animal-stabilized hyaluronic acid/dextranomer; OR, operating room; PNE, percutaneous nerve evaluation; PTNS, percutaneous tibial nerve stimulation; SNM, sacral nerve neuromodulation; VBS, vaginal bowel control system
